# Evaluation of Hydroxyl Ion Diffusion in Dentin and Injectable Forms and a Simple Powder-Water Calcium Hydroxide Paste: An in Vitro Study

**DOI:** 10.17795/jjnpp-14029

**Published:** 2014-06-14

**Authors:** Behrooz Eftekhar, Eskandar Moghimipour, Ebrahim Eini, Mansour Jafarzadeh, Narges Behrooz

**Affiliations:** 1 Department of Endodontics, Ahvaz Jundishapur University of Medical Sciences, Ahvaz, IR Iran; 2 Medicinal Plants Research Center, Ahvaz Jundishapur University of Medical Sciences, Ahvaz, IR Iran; 3 Student Research Committee, Ahvaz Jundishapur University of Medical Sciences, Ahvaz, IR Iran; 4 School of Dental, Ahvaz Jundishapur University of Medical Sciences, Ahvaz, IR Iran

**Keywords:** Calcium Hydroxide, Diffusion, Dentin

## Abstract

**Background:**

Intra canal medicaments are used to reduce the number of bacteria and reinfection in endodontic procedures. Calcium Hydroxide was introduced to endodontics by Herman as an intracanal antimicrobial agent.

**Objectives:**

The aim of this study was to present an injectable formulation of calcium hydroxide then compare the final pH of this new formulation with Metapaste and evaluate the effect of a mixture of Calcium Hydroxide powder with water on human extracted teeth.

**Patients and Methods:**

A total of 49 extracted human single-canal roots without caries and visible microcracks were included in this study. The teeth were decoronated and length of teeth was measured 1 mm anatomic apex. The canals were prepared using step-back technique. A cavity was created in the middle third of the buccal surface of all roots. The teeth were randomly divided into five groups: Group A (n = 15): In this group the root canals were filled with a mixture of calcium hydroxide powder and distilled water. Group B (n = 15): Included roots that were filled with Metapaste. Group C (n = 15): Root canals of this group were filled with new formulation of calcium hydroxide paste. Group D (negative control, n = 2): Included roots that were filled with a mixture of calcium hydroxide powder and distilled water. Group E (positive control, n = 2): Root canals of this group were filled with a mixture of calcium hydroxide powder and distilled water. Each tooth was immersed in a separate closed container with 4 mL saline for 2 weeks, pH of liquids were measured with an electrical pH meter after 7 and 14 days. The SPSS software (version 13) was used for data analysis. Analysis of variance (ANOVA) and Tukey tests were used for the statistical evaluation of results.

**Results:**

There was no significant difference at 7th day between the groups (P = 0.17) but at 14th day, a significant difference was observed between the groups (P = 0.04).

**Conclusions:**

The new formulation of calcium hydroxide with methylcellulose base has slower ionic dissolution, more durability and longevity of alkaline properties in comparison to combination of powder with distilled water and is comparable with other commercial products.

## 1. Background

Intra canal medicaments are used to reduce the number of bacteria and reinfection in the endodontic procedure (1, 2). Calcium hydroxide was introduced to endodontics by Herman as an intracanal antimicrobial agent (3). Previous studies have shown that calcium hydroxide may be the best available medicament among other agents to reduce the residual microbial flora (4). calcium hydroxide has a number of benefits including anti-fungal and anti-bacterial activities, mineralization activity and tissue dissolving capacity (5, 6). Antibacterial effect of intracanal medicament has a strong relationship with the direct contact between the medicament and microbial flora, thus calcium hydroxide should be placed deeply and compactly along the entire canal (7). The Hydroxyl ion released by the calcium hydroxide is considered to kill bacteria and the rate of killing can be influenced by the vehicle that is used (8). Three types of vehicle have been used (9):

1. Water soluble substances such as saline or ringer solution;
2. Viscous vehicles like glycerin or poly ethylene glycol;
3. Oil-based vehicles like olive oil or camphor.

There are several methods for inserting calcium hydroxide paste into the root canal such as MecaShaper, Lentulo spiral, injectable syringe, Gutta condenser and K-type ultrasonic file (1). Injectable paste syringes are time-saving but expensive. A few non-Iranian companies offer the formulations of drugs that can be injected by syringes. , also injectable type of calcium hydroxide is not made in Iran.

## 2. Objectives

The aim of this study was to present an injectable formulation of calcium hydroxide and then compare the final pH of this new formulation with Metapaste and a mixture of calcium hydroxide powder with water on the human extracted teeth.

## 3. Patients and Methods

A total of 49 extracted human single-canal roots without caries and visible microcracks were included in this study. We tried to select the teeth having relatively a similar root diameter. All teeth were washed with a soft brush and water and immersed for 24 hours in a solution of Formaldehyde (Armansina, Iran) in order to be disinfected, then were kept in normal saline until used. The teeth were decoronated and length of teeth was measured 1 mm below the anatomic apex. The canals were prepared using step-back technique. Preparation of the apical area was done by K-file up to size #30. Then flaring the canal was performed with #35 and #40 K-file (0.5 mm step back for each file). Afterwards, Gates Glidden drills size 2 and 3 (Maillefer-Switzerland) were used. Irrigation was performed between the use of each instrument with a solution of Sodium Hypochlorite 2.5%. Finally, after irrigating with %17 EDTA, all roots were washed with normal saline for three minutes. A cavity was created in the middle third of the buccal surface of all roots using a Wheel bur #038 and Depth Marker bur #016 (SWS Dental Diamond FG Burs-Switzerland). The initial form of the cavity was determined using a wheel bur and depth of cavity was raised to 1 mm in all areas by Depth marker bur. The final cavity was cylindrical with one mm depth and 3.8 mm diameter. Hydroxypropyl methyl cellulose (HPMC) with a concentration of 3% was used to produce gels. Water volume was calculated and Methylparaben was dissolved in it. Then HPMC was added gradually until a clear gel was obtained. An ultrasonic device was used for Homogenization. Calcium hydroxide with 43 wt% was gradually added to gel, as well as barium sulfate (7% wt) was added to it in several stages.

The teeth were randomly divided into five groups: group A (n = 15): In this group the root canals were filled with a mixture of calcium hydroxide powder and distilled water, group B (n = 15): Included roots that were filled with Metapaste, group C (n = 15): Root canals of the this group were filled with new formulation of calcium hydroxide paste, group D (negative control, n = 2): Included roots that were filled with a mixture of calcium hydroxide powder and distilled water, group E (positive control, n = 2): root canals of this group were filled with a mixture of calcium hydroxide powder and distilled water.

A lentulo (Maillefer-SwitzerLand) was used for all groups to insert paste into the canals and complete filling was assumed when paste got out of the apex. Orifice and apical foramen of each canal were covered with wax and all surfaces of roots except the cavity, which was created in the middle third of each root, were painted with nail varnish. In positive controls the nail varnish was not used and in negative controls all parts (root surfaces and the cavity) were completely covered by nail varnish. Each tooth was immersed in a separate closed container with 4 mL saline for 2 weeks. These containers were prewashed with hydrochloric acid 2.5%, pH of liquids were measured with an electrical pH meter after 7 and 14 days (pH meter electrode was placed in saline between measurements). The SPSS software (version 13) was used for data analysis. Analysis of variance (ANOVA) and Tukey tests were used for statistical evaluation of results.

## 4. Results

After seven days, maximum pH = 10.31 was in group A and minimum pH = 7.9 was in group B. The average of pH reading in the first, second and third groups were 8.52, 8.46, 8.96, respectively (Table 1). There was no significant difference at 7th day between the groups (P = 0.17) but at 14th day, a significant difference was observed between the groups (P = 0.04). There was a significant difference between groups one and three (P < 0.05) but no significant difference was observed between groups one and group two on the fourteenth day (P > 0.5). Also there was no significant difference between groups two and group three on the fourteenth day (P > 0.05).

On the seventh and fourteenth days the mean of pH readings in group one was 8.52 and 8.29, respectively, the Tukey test for two independent samples showed that the decrease in pH was not statistically significant (P > 0.05); the pH on fourteenth day compared to seventh day was decreased, however, the decrease was not statistically significant (P = 0.15). Mean of pH readings in group two on seventh and fourteenth days were 8.46 and 9.10 respectively and this increase was statistically significant (P < 0.05). Mean of pH readings in group three on seventh and fourteenth days were 8.96 and 9.61 respectively and this increase was significant statistically (P < 0.05).

## 5. Discussion

Effects of calcium hydroxide and hydroxyl ions in the tooth root environment have frequently been reported. Activity of the osteoclastic and inflammatory cells increases in acidic environment and can cause severe tissue degeneration (5, 10). Hydroxyl ion enhances osteogenic activity by changing the environment (11, 12). Also at the upper pH most of bacteria that are effective in endodontic infections cannot grow (13, 14). Thus, placing the combination of calcium hydroxide and a suitable carrier can boost the effects with creating a stable alkaline environment around the root. Different methods have been used to place calcium hydroxide in the canal, but in most studies Lentulo spiral was preferred, therefore in this study, it was used for calcium hydroxide placement (6). Various methods have been used to determine the level of pastes’ pH, including determination of pH by placing paste directly into a detector solution, determination of pH when paste was placed into the root canal and measuring the pH of solution in which teeth were immersed in (9, 15, 16). Calcium hydroxide paste with any carrier (water, methyl cellulose) creates higher pH when placed directly in a detector solution, but when paste was placed into the canal, level of pH was reduced due to the buffering effect of dentin or carbon containing gas (16, 17); also the hydroxyl ions can be absorbed by hydrate layer of hydroxyapatite which slows down their movement within the dentinal tubules (6).

As a carrier, water was used in the present study and the teeth had access to the saline only with simulated external root resorption, so hydroxyl ion transport through the apical foramen (the most effective route) and orifice was blocked. Biocompatibility of calcium hydroxide is indebted to low solubility in water but this property of calcium hydroxide prevents rapid increase of pH which is necessary to eliminate the bacteria (18). Hansen et al. found different pH at different levels of root. It may be related to the number and direction of dentinal tubules at each level (19). Perez et al. also showed that pH of dentin depends on the type, location and duration of calcium hydroxide application. To achieve as much similarity as possible between cases regarding the number, size, and orientation of the tubules, just one hole in the middle third of the buccal root surface of each tooth was created in our study (20).

Carriers affect not only ionization but also physicochemical properties and antimicrobial effects of calcium hydroxide. For water-based carriers such as saline and methylcellulose, the lower viscosity of paste, the more ionic resolution occurs (9). In the present study pH of groups 1, 2 and 3 were alkaline on the seventh day and there were no significant difference between these groups. The reason may be that all carriers are water-based. On the fourteenth day the pH in group 3 (new formulation) was significantly higher than group 1 (mixture of powder and water). Although carriers are water-based in both groups, it seems that new formulation keeps calcium hydroxide for a longer time at the optimal site due to higher molecular weight and jelly form of methylcellulose. This is in agreement with the study of Behnen et al. (21) in which it is mentioned that carboxymethylcellulose has a higher quality that allows easier diffusion of calcium hydroxide compared to other water-based solution with similar viscosity. In our study mean pH in group two and three was more than group one on the fourteenth day. Pacios et al. found that pH of different types of calcium hydroxide paste remains fixed regardless of the time intervals which is unlike our study (8). In the study of Perez et al. the pH was increasing for seven days in Hycal and this commercial paste had the highest pH compared to the mixture of calcium hydroxide and distilled water on the fourteenth day, which showed this gel-form paste has closer contact with canal walls and results in better canal filling (20). Nerwich et al. also observed a significant difference in the pH of root surfaces between Calset and a mixture of calcium hydroxide powder with saline that was in line with the present study (22). Similar to study of Safavi and Nakayama, in present study pH in group one slightly decreased on the fourteenth day compared to seventh day perhaps due to the dehydrating and faster ion dissolution (23). pH of group 2 and 3 increased on the 15th day in contrast with the study of Duarte et al. and in agreement with the study of Camargo et al.; the reason maybe that the fluid in which the teeth were immersed was not replaced in our study (24, 25). In all groups, the average pH was higher than 8 at all time intervals, thus hydroxyl ions can spread through dentinal tubules. Increase in pH of specimens was rarely higher than the pH required for the inactivation of *Enterococcus faecalis* (around pH = 11) (26, 27). In the present study, carriers other than distilled water increased the pH more at long term. These results were in contrast with study of Duarte et al. that showed the type of carrier is not an important factor in creating an alkaline environment (24), but when calcium hydroxide is agitated by ultrasonic into the canal, the greatest change in pH in the external root surface is recorded due to the greater penetration of powder particles of calcium hydroxide into the canal (28). In the study of Javidi et al. (29) Metapaste, Surpaste and Multical were compared for change of pH in the surrounding environment of root after 1, 24, 48 hour(s) and one week and it was concluded that Multical provided higher pH than Metapaste and Surpaste around the root surface. In addition, Metapaste has a significant pH decrease after 48 hours to one week, which was in contradiction with our study, in which Metapaste showed a steady pH increase upto fourteenth day. Average of pH changes created by Metapaste in this study was higher than our study on the seventh day. This discrepancy can be due to the removal of smear layer from the root surface in Javidi et al. study, which makes tubules more open in the inner root surface. Better penetration of hydroxyl ions and further increase in pH over the external surface of root could be one result. On the other hand, faster spread of calcium ion to outer surfaces and increase of its concentration can reduce the pH around the root (29). The new formulation of calcium hydroxide with methylcellulose base has slower ionic dissolution, more durability and longevity of alkaline properties in comparison to combination of powder with distilled water and is comparable with other commercial products.

**Table 1.** PH Values in Separate Groups and Time Points

Day	Number	Mean ± SD	Standard Error	Min-Max
**7th**				
Group 1	15	8.52 ± 0.96	0.24	7.14-10.31
Group 2	15	8.46 ± 0.76	0.19	7.09-9.90
Group 3	15	8.96 ± 0.59	0.15	8.07-9.89
Total	45	8.65 ± 0.80	0.11	7.09-10.31
**14th**				
Group 1	15	8.29 ± 1.03	0.26	7.04-10.90
Group 2	15	9.10 ± 1.46	0.37	7.02-11.35
Group 3	15	9.61 ± 1.58	0.40	7.16-11.22
Total	45	9.00 ± 1.46	0.21	7.02-11.35
